# Supplementary material for: Inhibitory maturation and ocular dominance plasticity in mouse visual cortex require astrocyte CB1 receptors
Source: iScience. 2024 Nov 17;27(12):111410. doi: 10.1016/j.isci.2024.111410 (PMC11647246; doi:10.1016/j.isci.2024.111410)
Supplement: Document S1. Figures S1 and Table S1 [file mmc1.pdf]

iScience, Volume *27*

Supplemental information

Inhibitory maturation and ocular dominance plasticity in mouse visual cortex require astrocyte CB1 receptors

Rogier Min, Yi Qin, Sven Kerst, M. Hadi Saiepour, Mariska van Lier, and Christiaan N. Levelt

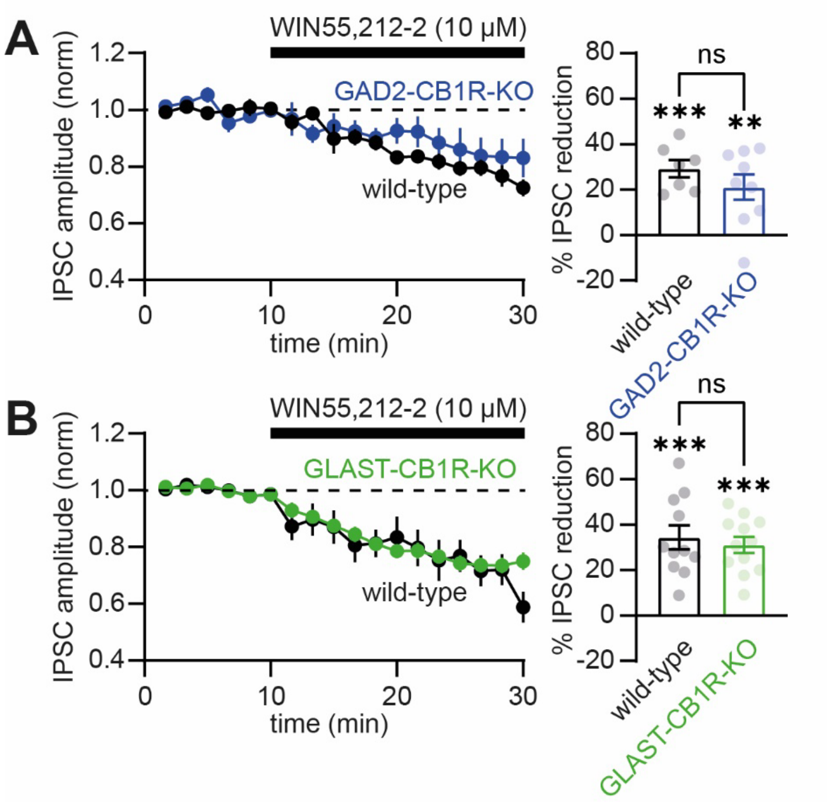

Figure S1. Synaptic depression by application of the synthetic CB1R agonist WIN55,212-2 is unaffected by removal of astrocyte or interneuron CB1 receptors, Related to Figure 3. (A) Left: Averaged time course of the IPSC amplitude in response to washin of WIN55,212-2 (10 µM), normalized to baseline. All recordings in GAD2-CB1R-KO mice (blue) and their wild-type littermates (black) are averaged. Right: Averaged amount of IPSC reduction (% reduction of the IPSC amplitude after application of WIN55,212-2) for all individual recorded neurons (GAD2-CB1R-KO IPSC reduction: 21.0±0.1%, n=9/N=5, IPSC reduction baseline vs after WIN55,212-2: *P*<0.05, paired t-test; wild-type littermates iLTD: 29.2±3.8% iLTD, n=7/N=3, IPSC amplitude baseline vs after WIN55,212-2: *P*<0.01; % reduction wild-type vs GAD2-CB1R-KO: *P*=0.27, unpaired t-test). (B) Same as in A, but now for GLAST-CB1R-KO mice (green) and their wild-type littermates (black; GLAST-CB1R-KO IPSC reduction: 29.4±3.5%, n=9/N=5, IPSC reduction baseline vs after WIN55,212-2: *P*<0.001, paired t-test; wild-type littermates iLTD: 34.4±5.3% iLTD, n=11/N=4, IPSC amplitude baseline vs after WIN55,212-2: *P*<0.001; % reduction wild-type vs GLAST-CB1R-KO: *P*=0.39, unpaired t-test). Error bars indicate SEM. *: P < 0.05; **: P < 0.01; ***: P < 0.001.

Table S1. Statistical details, Related to Figures 1-4.

FIGURE 2

	comparison	*test*	*P-value*	*one- or two-tailed?*	*Sum of Ranks (wt, KO)*	*Mann-Whitney U*
Figure 2A	steady state IPSC, littermate control vs GAD2-CB1R-KO	Mann-Whitney test	0,7069	two-tailed	276 , 354	140
Figure 2B	steady state IPSC, littermate control vs GLAST-CB1R-KO	Mann-Whitney test	0,0075	two-tailed	191 , 134	29

FIGURE 3

	comparison	*test*	*P-value*	*one- or two-tailed?*	*t, df*
Figure 3A	IPSC amplitude, baseline vs after iLTD induction, control	paired t-test	0,0002	two-tailed	t=4,366, df=28
	IPSC amplitude, baseline vs after iLTD induction, AM251	paired t-test	0,9221	two-tailed	t=0,09999, df=11
	% iLTD, control vs AM251	unpaired t-test	0,0023	two-tailed	t=3,264, df=39
Figure 3B	IPSC amplitude, baseline vs after iLTD induction, littermate control	paired t-test	<0.0001	two-tailed	t=4,719, df=39
	IPSC amplitude, baseline vs after iLTD induction, GAD2-CB1R-KO	paired t-test	0,0002	two-tailed	t=4,197, df=36
	% iLTD, littermate control vs GAD2-CB1R-KO	unpaired t-test	0,6464	two-tailed	t=0,4607, df=75
Figure 3C	IPSC amplitude, baseline vs after iLTD induction, littermate control	paired t-test	<0,0001	two-tailed	t=5,098, df=36
	IPSC amplitude, baseline vs after iLTD induction, GLAST-CB1R-KO	paired t-test	<0,0001	two-tailed	t=4,316, df=43
	% iLTD, littermate control vs GLAST-CB1R-KO	unpaired t-test	0,9056	two-tailed	t=0,1190, df=79

FIGURE S1

	comparison	*test*	*P-value*	*one- or two-tailed?*	*t, df*
Figure S1A	IPSC amplitude, baseline vs after WIN55,212-2 washin, littermate control	paired t-test	0,0006	two-tailed	t=6,468, df=6
	IPSC amplitude, baseline vs after WIN55,212-2 washin, GAD2-CB1R-KO	paired t-test	0,0053	two-tailed	t=3,791, df=8
	% reduction by WIN55,212-2, littermate control vs GAD2-CB1R-KO	unpaired t-test	0,2734	two-tailed	t=1,140, df=14
Figure S1B	IPSC amplitude, baseline vs after WIN55,212-2 washin, littermate control	paired t-test	0,0005	two-tailed	t=5,023, df=10
	IPSC amplitude, baseline vs after WIN55,212-2 washin, GLAST-CB1R-KO	paired t-test	<0,0001	two-tailed	t=7,544, df=11
	% reduction by WIN55,212-2, littermate control vs GLAST-CB1R-KO	unpaired t-test	0,6062	two-tailed	t=0,5234, df=21

FIGURE 4

	comparison	test	P-value	SS (Type III)	DF	MS	F (DFn, DFd)
Figure 4A	genotype (littermate control vs GAD2-CB1R-KO)	two-way ANOVA:					
	& monocular deprivation (no MD vs MD)	*Interaction*	0,9283	0,00008892	1	0,00008892	F (1, 17) = 0,008341
		Monocular Deprivation	<0,0001	0,3957	1	0,3957	F (1, 17) = 37,12
		Genotype	0,5307	0,004366	1	0,004366	F (1, 17) = 0,4096
		Residual		0,1812	17	0,01066	

	multiple comparisons:	test	P-value	Q	DF
	non MD:GAD2Cre-WT vs. non MD:GAD2Cre-KO	Tukey's multiple comparison	0,9807	0,5371	17
	non MD:GAD2Cre-WT vs. MD:GAD2Cre-WT	Tukey's multiple comparison	0,0023	6,136	17
	non MD:GAD2Cre-WT vs. MD:GAD2Cre-KO	Tukey's multiple comparison	0,0012	6,591	17
	non MD:GAD2Cre-KO vs. MD:GAD2Cre-WT	Tukey's multiple comparison	0,0052	5,575	17
	non MD:GAD2Cre-KO vs. MD:GAD2Cre-KO	Tukey's multiple comparison	0,0026	6,054	17
	MD:GAD2Cre-WT vs. MD:GAD2Cre-KO	Tukey's multiple comparison	0,9509	0,7477	17

	comparison	test	P-value	SS (Type III)	DF	MS	F (DFn, DFd)
Figure 4B	genotype (littermate control vs GAD2-CB1R-KO)	two-way ANOVA:					
	& monocular deprivation (no MD vs MD)	*Interaction*	0,022	0,0879	1	0,0879	F (1, 24) = 5,998
		Monocular Deprivation	0,0003	0,2697	1	0,2697	F (1, 24) = 18,40
		Genotype	0,0756	0,05056	1	0,05056	F (1, 24) = 3,450
		Residual		0,3517	24	0,01465	

	multiple comparisons:	test	P-value	Q	DF
	non MD:GLASTCre-WT vs. non MD:GLASTCre-KO	Tukey's multiple comparison	0,9772	0,5715	24
	non MD:GLASTCre-WT vs. MD:GLASTCre-WT	Tukey's multiple comparison	0,0006	6,508	24
	non MD:GLASTCre-WT vs. MD:GLASTCre-KO	Tukey's multiple comparison	0,34	2,42	24
	non MD:GLASTCre-KO vs. MD:GLASTCre-WT	Tukey's multiple comparison	0,0011	6,179	24
	non MD:GLASTCre-KO vs. MD:GLASTCre-KO	Tukey's multiple comparison	0,5407	1,911	24
	MD:GLASTCre-WT vs. MD:GLASTCre-KO	Tukey's multiple comparison	0,0205	4,471	24

	comparison	*test*	*P-value*	*SS (Type III)*	*DF*	*MS*	*F (DFn, DFd)*
Figure 4C	genotype (littermate control vs GAD2-CB1R-KO)	two-way ANOVA:					
	& monocular deprivation (no MD vs MD)	*Interaction*	0,0014	1,025	1	1,025	F (1, 434) = 10,31
		Monocular Deprivation	<0,0001	7,207	1	7,207	F (1, 434) = 72,44
		Genotype	0,0055	0,7742	1	0,7742	F (1, 434) = 7,781
		Residual		43,18	434	0,0995	
	multiple comparisons:	*test*	*P-value*	*Q*	*DF*		
	non MD:GLASTCre-WT vs. non MD:GLASTCre-KO	Tukey's multiple comparison	0,9886	0,453	434		
	non MD:GLASTCre-WT vs. MD:GLASTCre-WT	Tukey's multiple comparison	<0,0001	11,64	434		
	non MD:GLASTCre-WT vs. MD:GLASTCre-KO	Tukey's multiple comparison	0,0002	5,99	434		
	non MD:GLASTCre-KO vs. MD:GLASTCre-WT	Tukey's multiple comparison	<0,0001	10,84	434		
	non MD:GLASTCre-KO vs. MD:GLASTCre-KO	Tukey's multiple comparison	0,001	5,34	434		
	MD:GLASTCre-WT vs. MD:GLASTCre-KO	Tukey's multiple comparison	0,0005	5,625	434		
